# Photocatalytic Degradation of Rhodamine B Dye and Hydrogen Evolution by Hydrothermally Synthesized NaBH_4_—Spiked ZnS Nanostructures

**DOI:** 10.3389/fchem.2022.835832

**Published:** 2022-04-14

**Authors:** Theopolina Amakali, Aleksandar Živković, Michael E. A. Warwick, Daniel R. Jones, Charles W. Dunnill, Likius S. Daniel, Veikko Uahengo, Claire E. Mitchell, Nelson Y. Dzade, Nora H. de Leeuw

**Affiliations:** ^1^ Department of Physics, Chemistry and Material Science, University of Namibia, Windhoek, Namibia; ^2^ School of Chemistry, Cardiff University, Cardiff, United Kingdom; ^3^ Department of Earth Sciences, Utrecht University, Utrecht, Netherlands; ^4^ Energy Safety Research Institute, Swansea University, Swansea, United Kingdom; ^5^ Multidisciplinary Research, Centre for Research Service, University of Namibia, Windhoek, Namibia; ^6^ School of Chemistry, University of Leeds, Leeds, United Kingdom

**Keywords:** ZnS, photocatalysis, rhodamine B degradation, NaBH4 adsorption, density functional theory, HSE06, defect states, sulphur vacancies

## Abstract

Metal sulphides, including zinc sulphide (ZnS), are semiconductor photocatalysts that have been investigated for the photocatalytic degradation of organic pollutants as well as their activity during the hydrogen evolution reaction and water splitting. However, devising ZnS photocatalysts with a high overall quantum efficiency has been a challenge due to the rapid recombination rates of charge carriers. Various strategies, including the control of size and morphology of ZnS nanoparticles, have been proposed to overcome these drawbacks. In this work, ZnS samples with different morphologies were prepared from zinc and sulphur powders via a facile hydrothermal method by varying the amount of sodium borohydride used as a reducing agent. The structural properties of the ZnS nanoparticles were analysed by X-ray diffraction (XRD), scanning electron microscopy (SEM), and X-ray photoelectron spectroscopy (XPS) techniques. All-electron hybrid density functional theory calculations were employed to elucidate the effect of sulphur and zinc vacancies occurring in the bulk as well as (220) surface on the overall electronic properties and absorption of ZnS. Considerable differences in the defect level positions were observed between the bulk and surface of ZnS while the adsorption of NaBH_4_ was found to be highly favourable but without any significant effect on the band gap of ZnS. The photocatalytic activity of ZnS was evaluated for the degradation of rhodamine B dye under UV irradiation and hydrogen generation from water. The ZnS nanoparticles photo-catalytically degraded Rhodamine B dye effectively, with the sample containing 0.01 mol NaBH_4_ being the most efficient. The samples also showed activity for hydrogen evolution, but with less H_2_ produced compared to when untreated samples of ZnS were used. These findings suggest that ZnS nanoparticles are effective photocatalysts for the degradation of rhodamine B dyes as well as the hydrogen evolution, but rapid recombination of charge carriers remains a factor that needs future optimization.

## 1 Introduction

Research on photocatalysis has increased exponentially in recent years and a multitude of materials have been synthesized and explored as photocatalysts for the degradation of organic pollutants in wastewater as well as the photocatalytic degradation of water to generate hydrogen for use as a clean fuel (Lee and Wu, 2017; Cao et al., 2020). Transition metal oxides and chalcogenides including titanium (IV) oxide (TiO_2_), cadmium selenide (CdSe), cadmium sulphide (CdS), zinc oxide (ZnO), and zinc sulphide (ZnS) are some of the semiconductor materials that have been widely investigated for photocatalytic applications (Haque et al., 2018; Zhuang et al., 2021). Semiconductor-mediated photocatalysis owes its popularity to a number of advantages including low cost, simplicity, and ease of preparation. Titanium (IV) oxide (TiO_2_) has received the most attention, but despite its popularity, its application is hindered by low quantum efficiency and photo-corrosion (Gopinath et al., 2020).

ZnS has attracted much attention as a potential and effective photocatalyst for applications in organic pollutant degradation (Dong et al., 2013; Ma et al., 2016; Ye et al., 2018) and in the hydrogen evolution reaction (HER) via water splitting (Puentes-Prado et al., 2020; Samaniego-Benitez et al., 2021; Xiong et al., 2021). Zinc sulphide is polymorphous with a cubic zinc blende (sphalerite) structure that is more stable at low temperature and a hexagonal wurtzite structure which commonly forms at high temperatures. It has one of the richest and most diverse morphologies, exhibiting different properties that allow its use in various applications, including nanowires (Cao et al., 2014), nanorods (Palanisamy et al., 2020), nanoflowers (Bai et al., 2014), nanospheres (Feng et al., 2020), nanotubes (Wang et al., 2017), nanobelts (Ham and Jang, 2018), and nanosheets (Fang et al., 2011). For example, nanorods, nanoarrays, and nanoflowers exhibit light-trapping effects that enhance light absorption which in turn results in increased photocatalytic activity (Bhushan and Jha, 2020; Ren et al., 2021; Aulakh et al., 2022). A review by Fang et al. (2011) have provided a detailed survey of research activities related to ZnS nanostructures with various morphologies. In this review, the interaction between synthesis conditions and the diverse nanoscale morphologies of ZnS was elucidated as well as their application in various functional devices. In another review, Lee et al. (2017) have presented a comprehensive discussion on band gap engineering studies, synthetic methods and photocatalytic applications of ZnS nanocrystalline semiconductors. The authors also gave a detailed explanation of possible reaction mechanisms for organic pollutant degradation and the photocatalytic hydrogen evolution.

Zinc sulphides show good photocatalytic activity under UV irradiation, high theoretical efficiency, and they rapidly generate charge carriers upon photoexcitation. In addition, ZnS has excellent chemical stability against oxidation and hydrolysis and these properties are maintained when the particle size is reduced to nano levels. However, its main shortcoming is the large negative potential value and limited visible light absorption due to its wide band gap, which is 3.54 and 3.91 eV for the cubic and hexagonal structures, respectively.

One strategy used to improve the light-harvesting properties of ZnS nanostructures is by introducing surface defects, which will serve as adsorption sites where charge transfer to an adsorbed species prevents charge recombination of photogenerated charge carriers (Zhang et al., 2014; Wang et al., 2015a; Dong et al., 2017; Yuan et al., 2019; Liu et al., 2020; Ghosh et al., 2021). These defects however should be controlled, because excessive defects will act as a trap for charge carriers, thereby decreasing the photocatalytic activity.

The preparation method, process parameters, and the synthesis conditions employed during the preparation of ZnS nanostructures play a crucial role in the modification of their surface characteristics, particle size, morphology, and structure, hence affecting the resultant photocatalytic activity (Zhang et al., 2014; Ebrahimi and Yarmand, 2019). Yin et al. (2016) prepared cubic ZnS particles of controlled size and morphologies with enhanced photocatalytic activities by simply manipulating the molar ratio of Zn/S. A degradation efficiency of more than 96% was achieved in just 24 min when the Zn/S molar ratio equalled 1:2. Sabaghi et al. (2018), using a simple hydrothermal and reflux methods and varying the ratio of precursor materials (zinc acetate dihydrate/thioacetamide) as well as changing the reaction time managed to synthesize cubic sphalerite ZnS nanoparticles of various morphologies and size, e.g., sheetlike, flowerlike, and sphere. All parameters investigated, such as the method of synthesis, mole ratio, and reaction time influenced the photocatalytic degradation of Reactive blue 21 pigment. The hydrothermal method using precursor materials with a molar ratio equal to 1:1 and carried out for 12 h showed the greatest efficiency, completely (100% degradation) removing the pigment after 240 min. Various other works have investigated the effect of synthesis conditions on the photocatalytic performance of ZnS nanoparticles, without the addition of a co-catalyst, but summarising the details of these is beyond the scope of this article (Dong et al., 2013; Zhang et al., 2014; Ma et al., 2016; La Porta et al., 2017; Lee and Wu, 2017; Liu et al., 2020).

The reduction ability of borohydrides has attracted attention, owing to their ability to generate a large number of nucleating cores, thereby causing a disordered growth process with greater diversity in nanoparticle size (Saravanakumar et al., 2017). Wang et al. (2015b) prepared a ZnS photocatalyst with controlled sulphur vacancies by adding small amounts of sodium borohydride (NaBH_4_) and they observed an increase in the photocatalytic activity of the ZnS photocatalyst (0.5 g) towards hydrogen evolution with increasing amount of NaBH_4_, which first reached a maximum before decreasing again with a further increase in NaBH_4_ concentration.

In the present work, ZnS nanoparticles were synthesized using NaBH_4_ as a reducing agent and the sample was evaluated for the photocatalytic degradation of rhodamine B dye as well as hydrogen evolution from a water splitting reaction. Moreover, theoretical investigations at a hybrid-DFT approximation level were undertaken to study the effect of vacancies on the structural and electronic properties of ZnS. In addition, the adsorption of NaBH_4_ atop a ZnS surface was studied and the energetics as well as influence on the electronic properties elucidated.

## 2 Experimental and Computational Details

### 2.1 Materials

All chemical reagents (Zn powder, sublimed sulphur, NaOH, NaBH_4_, Na_2_S_9_ H_2_O, Na_2_SO_3_) used in this study were analytical reagents (AR) procured from Sigma- Aldrich and used as received without any further purification.

### 2.2 Synthesis of ZnS Samples

Zinc sulphide nanoparticles of different morphologies were synthesized via a hydrothermal route. The precursor solution was prepared by mixing equal amounts (0.0205 mol) of zinc and sulphur powders in 70 ml of concentrated NaOH (21 M) solution. After the suspension was cooled to room temperature, different amounts of NaBH_4_ (0 mol, 0.003 mol, 0.005 mol, 0.01 mol, 0.02 mol) were added and the mixture was stirred at room temperature for 2 h. The suspension was transferred to a 125 ml Teflon-lined autoclave, sealed, and placed in a furnace at 230°C for 12 h. The product was collected after centrifuging and washing several times with deionized water. Finally, the product was dried in an oven at 40°C for 72 h.

### 2.3 Characterization

For structural characterization of the samples, X-ray diffraction (XRD) patterns were recorded using a Bruker D8 Advance X-ray diffractometer with Cu-Kα radiation (*λ* = 1.5406 Å) with the scanning angle ranging from 10° to 80° to determine the phase composition. The morphology of the product was analysed by Field Emission scanning electron microscopy (FE-SEM) using a Hitachi S4800 FE-SEM operating at an accelerating voltage of 10 kV and an emission current of 10 μA.

Surface chemical analysis was achieved through X-ray photoelectron spectroscopy (XPS) performed on a Thermo Scientific K-Alpha^+^ spectrometer with a monochromated Al K-alpha source (1,486.68 eV) operating at 72 W; spectra were obtained from an elliptical 800 μm × 400 μm analysis area and charge neutralisation was applied throughout. Core spectra were acquired using a dwell time of 500 m and a pass energy of 20 eV, and all binding energies were carbon-corrected by referencing the C 1s peak to a standard value of 284.8 eV. CasaXPS software was employed to carry out spectral deconvolution, for which Gaussian-Lorentzian components with 30% Lorentzian character were fitted to the measured data above a Shirley-type background function. Physically realistic fits were obtained by imposing a 2:1 area ratio and equal full-widths-at-half-maximum for each pair of 2p_3/2_ and 2p_1/2_ components, whilst the spin orbit separation between them was constrained to characteristic values of 23.00 eV (Wang et al., 2015b; Kwoka et al., 2020) and 1.16 eV (Sarkar et al., 2018) for Zn 2p and S 2p spectra, respectively. After calibrating for the transmission function of the spectrometer and applying mean free path correction, the total measured area of each deconvolved spectrum was divided by the relevant relative sensitivity factor to determine the surface atomic composition of a given sample.

For optical properties, the UV-Vis diffuse reflectance spectra were recorded on a CARY 100 UV-Vis spectrophotometer of Agilent technology in the region between 200 and 700 nm. The band gaps of the samples were calculated from diffuse reflectance using the Kubelka-Munk function by plotting [hυF(r)]^2^ vs. hυ, where hυ is the photon energy, f(r) is the Kubelka-Munk function and extending the tangent of the curve to the hυ axis. Room temperature photoluminescence (PL) spectra of the samples were measured using a 7.8 Mw Melles-Griot He-Cd laser (325 nm) with an incident power density of 2.3 × 10^6^ mW m^2^) over a wavelength region of 350–750 nm.

### 2.4 Photocatalytic Performance

The photocatalytic performance was evaluated through the degradation of rhodamine B dye (4 mg/ml) under UV irradiation at room temperature in the presence of a ZnS photocatalyst (20 mg). The mixture was first stirred in the dark for 30 min to achieve the absorption-desorption equilibrium. It was then exposed to the UV light over various durations of time. The change in concentration after each duration was recorded on the UV-vis spectrophotometer in the region of 400–650 nm.

The photocatalytic activity of the prepared ZnS samples was evaluated for hydrogen generation via the water splitting reaction under visible light irradiation. Typically, 20 mg of the sample was added to 20 ml aqueous solution containing 0.35 M Na_2_S and 0.25 M Na_2_SO_3_ as a sacrificial reagent. This was placed in a glass vial connected to a closed gas circulation and evacuation system. The reaction solution was kept under continuous magnetic stirring and the irradiation of the system was achieved by using a 100 W LED lamp. An online gas chromatograph (782 A GC system of Agilent Technology) equipped with thermal conductive detector (TCD) was used to determine the generated gas products using argon as a carrier gas.

### 2.5 Computational Details

Spin-polarized density functional theory (DFT) calculations were performed using the all-electron (AE) code CRYSTAL (2017 release) (Pascale et al., 2004; Zicovich-Wilson et al., 2004; Dovesi et al., 2017; Dovesi et al., 2018). The short-range corrected range separated hybrid Heyd−Scuseria−Ernzerhof (HSE06, unmodified) (Heyd et al., 2003; Heyd and Scuseria, 2004; Heyd et al., 2006) functional was employed together with basis sets based on Gaussian-type orbitals (GTOs). The following GTO basis sets were employed: for Zn constructed and optimized by Jaffe et al. (1994), (Jaffe and Hess, 1993), for S from Lichanot et al. (1993), (Zagorac et al., 2018), for Na from Dovesi et al. (1991), for B the 6–21G* by John Pople (Orlando et al., 1990), and for H by Dovesi et al. (1984). One additional *d*-function (with an exponent of 0.206368) was added to the Zn basis set, while the remaining basis sets were taken without further modification. In the structural optimizations, both the atomic positions and the lattice constants were fully optimized within the constraints imposed by the space group symmetry. The reciprocal space was sampled using 11 × 11 × 11 and 3 × 3 × 1 Monkhorst-Pack type k-point grids for ZnS bulk and a 3 × 2 × 1 supercell of the (220) surface of ZnS, respectively (Monkhorst and Pack, 1976). For the evaluation of the Coulomb and exchange integrals, the default tolerance factor values of 6, 6, 6, 6, and 12 were used (TOLINTEG). Long range dispersion corrections were included using the semiempirical D3 approach of Grimme et al. (2010) with Becke-Johnson damping (Grimme et al., 2011; Grimme et al., 2016). Band structure calculations were performed on optimized geometries along high-symmetry directions obtained using the SeeK-path interface (Hinuma et al., 2017; Togo and Tanaka, 2018). Graphical drawings were produced using VESTA (Momma and Izumi, 2011). Excitonic and spin-orbit coupling effects were not considered.

The formation energy $\left(E^{f}\right)$ of defects present in the system was defined as:

$$E^{f}\left(D\right)=E_{tot}\left(D\right)-E_{tot}\left(H\right)-\sum_{i}n_{i}\mu_{i},$$

where $E_{tot}\left(D\right)$ and $E_{tot}\left(H\right)$ denote the respective total energies of the system with and without the induced defect. The value $n_{i}$ represents the number of atoms of element i that are added ( $n_{i}>0$ ) or removed ( $n_{i}<0$ ) from the supercell to form the defect, and $\mu_{i}$ (which can be written as $\mu_{i}=\mu_{i}^{elem}+△\mu_{i}$ ) is the chemical potential of element i in its standard phase, referenced to the total energy of the elementary phases at 0 K [i.e., $\mu_{i}^{elem}$ of Zn(s) and S_8_(g)]. In the region of phase space where ZnS is stable, the chemical potentials are further constrained by the equilibrium condition:

$$△\mu_{Zn}+△\mu_{S}=△H_{f}\left(ZnS\right).$$

The formation enthalpy of ZnS is calculated at −2.307 eV, which is in good agreement with the experimental value of −2.136 eV (Richards, 1959). Together with the limit that the chemical potential of the atoms in the reservoir must be lower than the elemental reference energies ( $△\mu_{Zn}\leq 0$ and $△\mu_{S}\geq 0$ ), the upper bound for the chemical potentials can be obtained: ( $△\mu_{Zn}=0$ eV, $△\mu_{S}=-2.307eV$ ) in the Zn-rich environment and ( $△\mu_{Zn}=-2.307$ eV, $△\mu_{S}=0$ eV) in the S-rich environment.

The surface was modelled as a two-dimensional slab, and no three-dimensional periodicity was imposed. This means that there is no parameter for the vacuum thickness needed. To characterise the surface, the surface energy (γ) as a measure of the thermodynamic stability has been calculated through the following expression:

$$\gamma =\frac{E\left(n\right)-n\cdot E_{bulk}}{2\cdot A},$$

where E(n) is the energy of the slab containing n -layers, $E_{bulk}$ the energy of the bulk, and A the area of one side of the slab.

The binding energy of NaBH_4_ $\left(E_{b}\right)$ was calculated from the fully relaxed geometries, through the following expression:

$$E_{b}=E_{slab+ads}-\left(E_{slab}+E_{ads}\right),$$

where $E_{slab+ads}$ is the total energy of the slab interacting with the absorbate, while $E_{slab}$ and $E_{ads}$ denote the total energies of the pristine surface and isolated adsorbate, respectively. Within this definition of the binding energy, a negative value indicates a favourable exothermic process, while a positive energy corresponds to an endothermic process. When using atomic-centred localised basis sets, the binding energy is affected by the basis set superposition error (BSSE), which corresponds to an artificial increase (spurious extra-binding that mimics the dispersion energy) in the computed binding energy because the basis set of the final system is larger than that of the component subsystems. One way to estimate the BSSE is using the counterpoise (CP) method, where one recalculates $E_{slab}$ and $E_{ads}$ by supplementing the basis set of each subsystem with all the basis functions of the other without their electrons and nuclei (referred to as “ghost functions”). The BSSE is a positive quantity and equals to:

$$BSSE=\left(E_{ads|system}-E_{ads|system}^{G}\right)+\left(E_{slab|system}+E_{slab|system}^{G}\right),$$

where $E_{ads|system}^{G}$ and $\;E_{slab|system}^{G}$ denote the energies of the adsorbate and surface calculated respectively in the presence of the “ghosted atoms” of the surface and of the adsorbate (i.e., including the extra basis set of the surface or the adsorbate). Taking into account the BSSE, the CP-corrected binding energy $\left(E_{b}^{CP}\right)$ reads (Boys and Bernardi, 1970; Scaranto et al., 2011):

$$E_{b}^{CP}=E_{b}+BSSE.$$

## 3 Results and Discussion

### 3.1 Characterization

**FIGURE 1:**
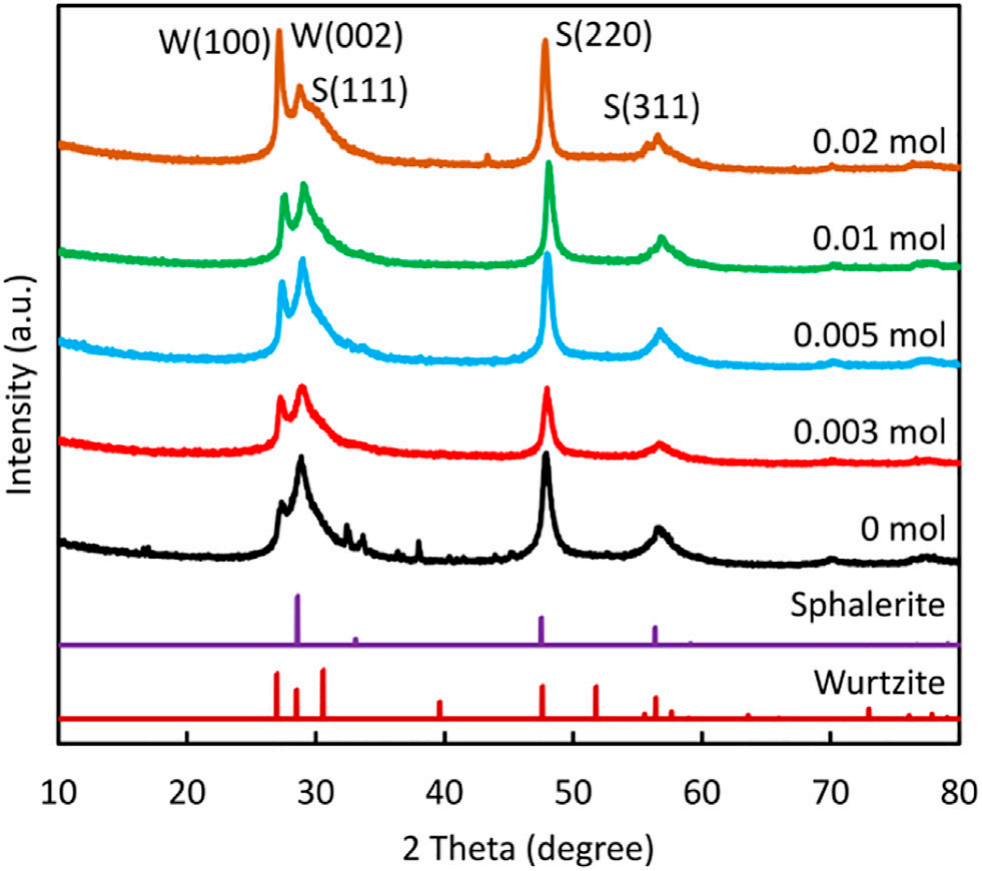
XRD patterns of ZnS prepared using different amounts of NaBH_4_.

Figure 1 shows the XRD pattern of the ZnS nanostructures prepared using different amounts of NaBH_4_ in NaOH. The pattern matched that of the low temperature cubic sphalerite ZnS nanostructure (data file card: COD 5000088). The position of the two main diffraction peaks obtained for all samples is the same and were recorded at 2*θ* values (48.11°) and (57.11°), corresponding to the (220) and (311) crystal plane respectively. In addition, there is a strong -sharp peak and an overlapping peak between 26.94° and 30.4°. Strong and sharp peak patterns at 2*θ* value (27.2°) can be indexed as the (100) reflection of wurtzite ZnS, and the broader peaks next to it are the overlapping peaks of wurtzite (002) and sphalerite (111). Although the (111), (220), and 311) peaks of the sphalerite ZnS occupy the same position as the (002), (110), and (112) of the ZnS wurtzite structure in the XRD pattern, the missing peaks in the wurtzite standard pattern suggest that a sphalerite ZnS structure, rather than the wurtzite structure, dominates in the nanoparticles.

The crystallite size was calculated from the sharp and intense peak of 48.11° using the Scherrer equation:

$$D=\frac{K\lambda }{\beta \cos\theta },$$

where K is a constant (k = 0.89), λ is the wavelength, β is the full width value at half maximum of the intensity (FWHM) and *θ* is the Bragg angle corresponding to the (220) peak. The values of the crystallite size were 12.1 nm, 12.5 nm, 12.4 nm, 13.9 nm, and 8.04 nm corresponding to ZnS samples prepared with 0 mol, 0.003 mol, 0.005 mol, 0.01 mol, and 0.02 mol of NaBH_4_ respectively. The crystallite size grew slightly with an increase in added NaBH_4_, reaching a maximum (13.9 nm) for 0.01 mol of NaBH_4_ and then decreasing to 8.04 nm for 0.02 mol of NaBH_4_.

**FIGURE 2:**
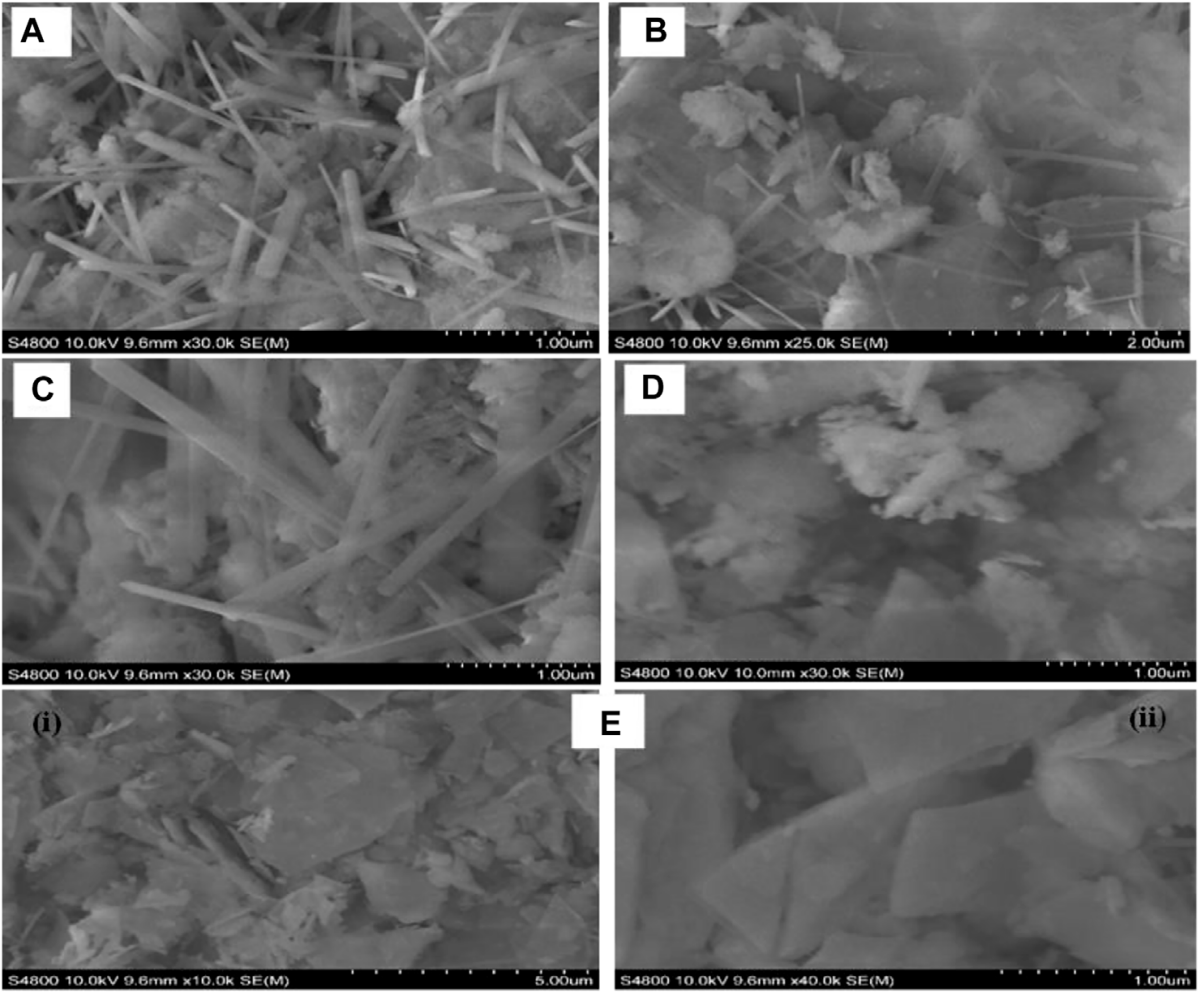
SEM images of ZnS prepared with different amounts of NaBH_4_ **(A)** 0 mol **(B)** 0.003 mol **(C)** 0.005 mol **(D)** 0.01 mol **(E)** 0.02 mol.

The SEM morphologies obtained for the ZnS nanostructures differ with the amount of added NaBH_4_ (Figures 2A–E). At lower NaBH_4_ concentrations (0–0.005 mol NaBH_4_), the nanoparticles are composed of a mixture of nanorods and agglomerates without defined shape. As the concentration of NaBH_4_ increases (0.01 mol NaBH_4_), the nanorods disappeared and the nanoparticles became featureless agglomerates with no particular shape at 0.01 mol NaBH_4_. However, when the ZnS were synthesized with the addition of 0.02 mol NaBH_4_, rectangular slabs-like nanostructures were obtained as shown in Figure 2E (i) at low magnification and Figure 2E (ii) at high magnification. The different morphologies can be attributed to the effect of NaBH_4_ that was added.

NaBH_4_ has been shown to induce the formation of long size nanorods in, e.g., metal-doped ferrites (Ni-ferrites) due to the clustering of NaBH_4_ around the metal ion (Saravanakumar et al., 2017). This effect was not observed in this study and the shape of the nanostructure was rather distorted by the increased concentration of NaBH_4_. It is reported that borohydrides have the ability to generate a high concentration of nanocrystals with small nuclei in a short time, forming a large number of cores and disfavouring the growth process of nanostructures (Oliveira et al., 2020). This results in a disordered growth process which explains the featureless agglomerates formed with increasing concentration of NaBH_4_. Other factors however, such as such as pH (not investigated in this study) could have an influence on the shape of the nanostructures and as such should be ruled out without further investigation.

**FIGURE 3:**
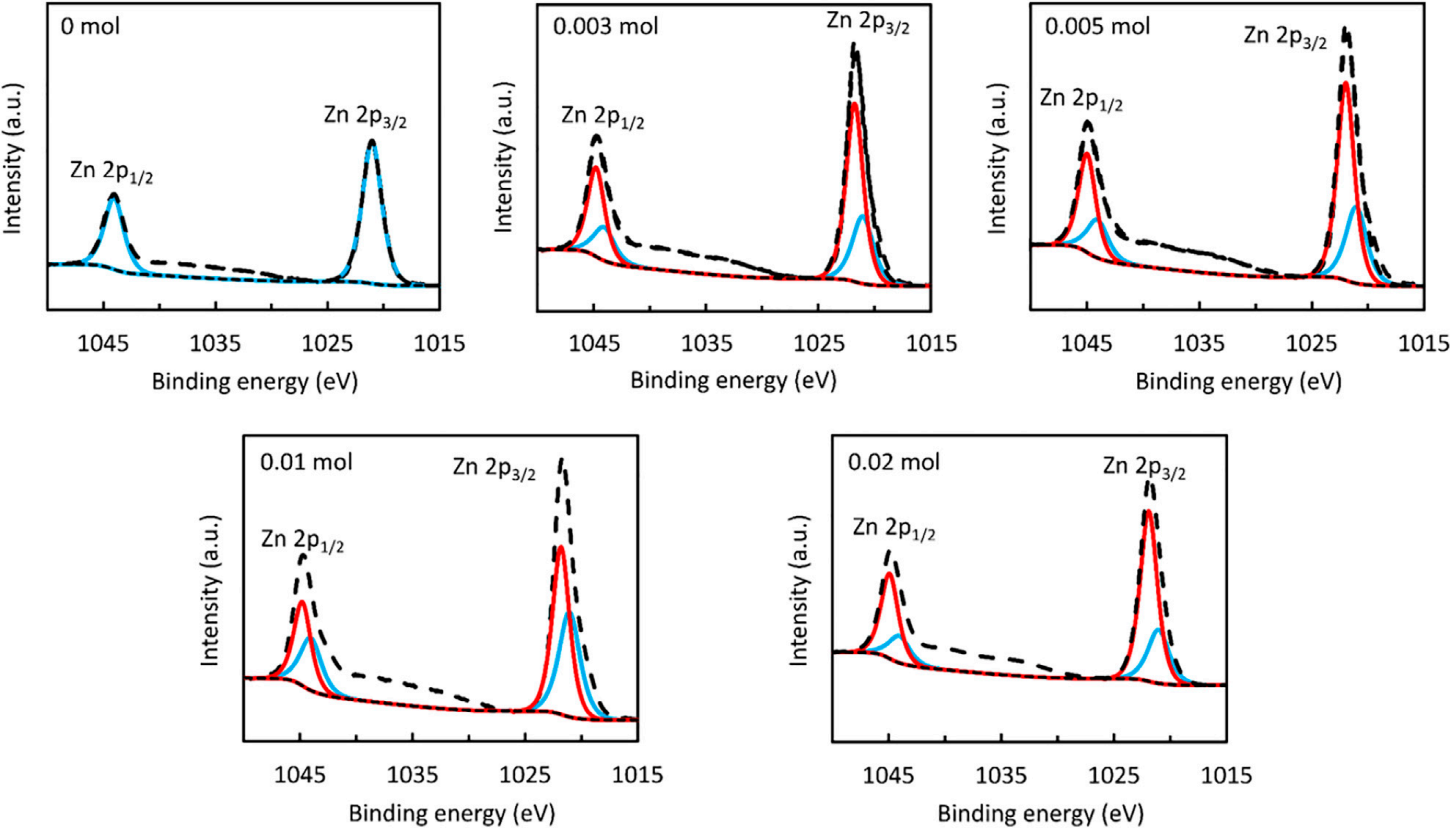
Zn (2p^3^) XPS spectrum of ZnS nanostructures prepared using different amounts of NaBH_4_.

For the sample that was synthesized without the addition of NaBH_4_, the Zn 2p XPS spectrum is well-represented by a single doublet with 2p_1/2_ and 2p_3/2_ peaks centred at binding energies of 1,044.1 eV and 1,021.1 eV, respectively, as shown in Figure 3. Whilst a similar doublet provides the predominant contribution to the corresponding spectra of the NaBH_4_-treated samples, the asymmetric form of each peak is consistent with a second doublet offset from the first by 0.7–0.9 eV. Since the chemical shifts between Zn 2p spectra associated with different Zn electronic environments are characteristically on the order of 100 meV, even when considering disparate oxidation states of Zn (Biesinger et al., 2010), the comparatively large separation of the Zn 2p doublets suggests that differential charging occurred within the NaBH_4_-synthesized samples, in turn indicating that they contained a mixture of materials with differing electrical conductivities. It is possible, for instance, that the inclusion of NaBH_4_ during ZnS synthesis yielded discrete particles of ZnO, which is considerably more conductive than ZnS (Zafar et al., 2021). One should note that as a highly surface-sensitive technique, XPS cannot detect species at a distance greater than ∼ 10 nm from the sample surface, in contrast to bulk characterisation procedures such as XRD. For this reason, the high bulk purity of ZnS evident from the XRD data shown in Figure 1 does not preclude the possibility that ZnO nanoparticles existed at the ZnS surface, thereby influencing the Zn 2p spectra but nevertheless having negligible impact on the diffraction properties of the bulk material.

**FIGURE 4:**
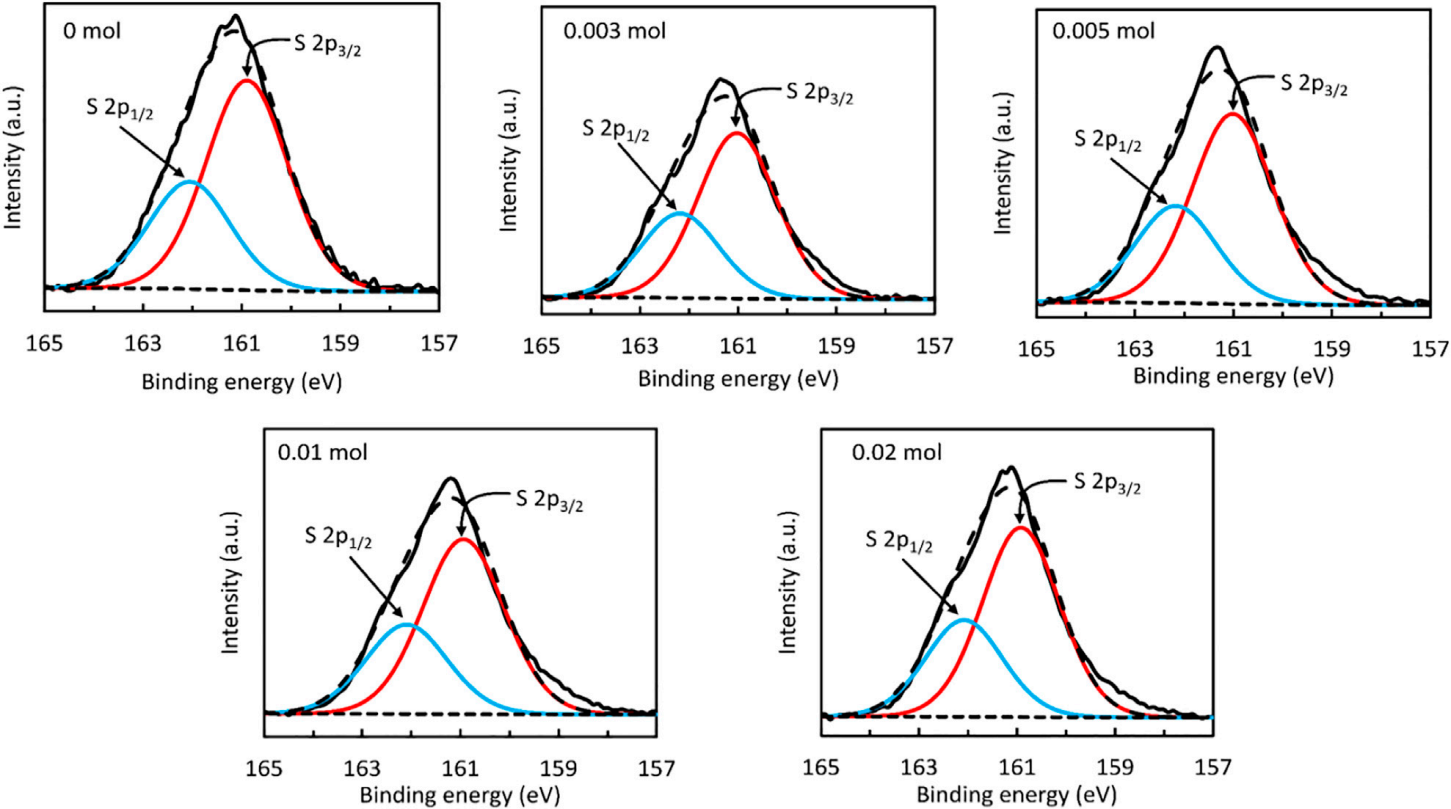
S (2p) XPS spectrum of ZnS nanostructure prepared using different amounts of NaBH_4_.

To address the chemical environments of S within the ZnS samples, deconvolved S 2p spectra are presented in Figure 4. As it is possible in all cases to use a single doublet to reproduce the form of the S 2p peak, one may infer that each sample surface comprised only one S-containing compound in significant quantities. Furthermore, the 2p_1/2_ and 2p_3/2_ components are centred at binding energies of 162.1–162.2 eV and 160.9–161.0 eV, respectively, which are consistent with the S 2p peak positions typically reported for ZnS (Tran et al., 2022).

Finally, estimates of the S/Zn surface atomic ratio were obtained from the measured areas of the Zn 2p and S 2p spectra to provide insight into the relative proportions of S-containing and S-free compounds within each sample. As shown in Table 1, the S/Zn ratio was found in all cases to have a value in the range 0.84–0.88, which is significantly lower than the value of unity expected for pristine ZnS. In accordance with discussions regarding the Zn 2p spectra, this discrepancy may be attributed to the presence of S-free species such as ZnO and Zn(OH)_2_ at the ZnS surface, with the samples containing similar quantities of these compounds.

**TABLE 1:** Chemical composition of ZnS samples measured via XPS.

Sample	S (at%)	Zn (at%)	S/Zn
0 mmol NaBH_4_	32.34	36.94	0.875
3 mmol NaBH_4_	36.33	42.28	0.859
5 mmol NaBH_4_	35.58	42.45	0.838
10 mmol NaBH_4_	36.36	42.61	0.866
20 mmol NaBH_4_	35.50	10.83	0.869

**FIGURE 5:**
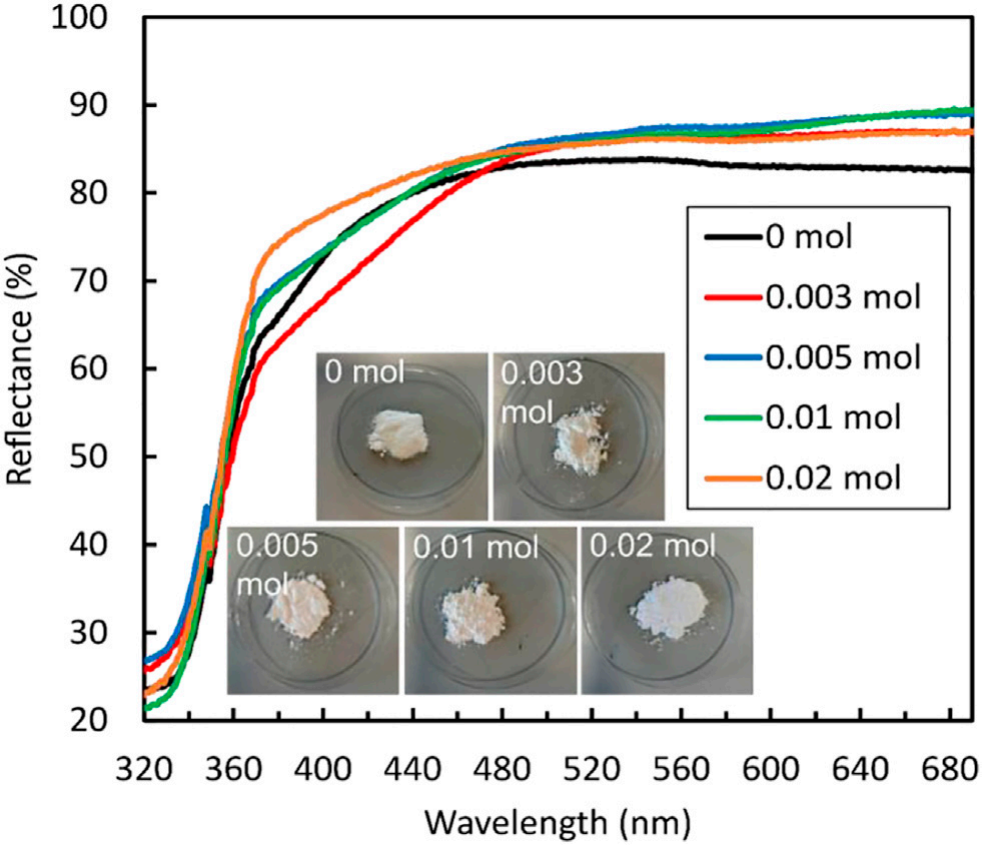
UV-Vis diffuse reflectance spectra of ZnS prepared using different amounts of NaBH_4_, together with the images of the samples.

The optical properties of the ZnS samples were analysed by UV-vis reflectance spectroscopy to evaluate their light absorption ability. As shown in Figure 5, the addition of NaBH_4_ slightly increased the diffuse reflectance until 0.01 mol but then decreased again when the highest concentration of 0.02 mol NaBH_4_ was reached. However, the samples of ZnS nanoparticles showed very little variation in band gap energy value 3.54 eV, 3.54 eV, 3.55 eV, 3.55 eV, and 3.57 eV, for the nanoparticles prepared with 0 mol, 0.003 mol, 0.005 mol, 0.01 mol, and 0.02 mol NaBH_4_, respectively. The decreasing crystallite size of the sample prepared with 0.02 mol of NaBH_4_ could be the reason for the slight blue shift in optical band gap observed for this sample. These results are in conflict with what is reported by Wang et al. (2015b) who reported a decreasing optical band gaps with increasing amount of NaBH_4_, although other factors such as residual internal stress could be the cause of such discrepancies or trend (Ma et al., 2016).

**FIGURE 6:**
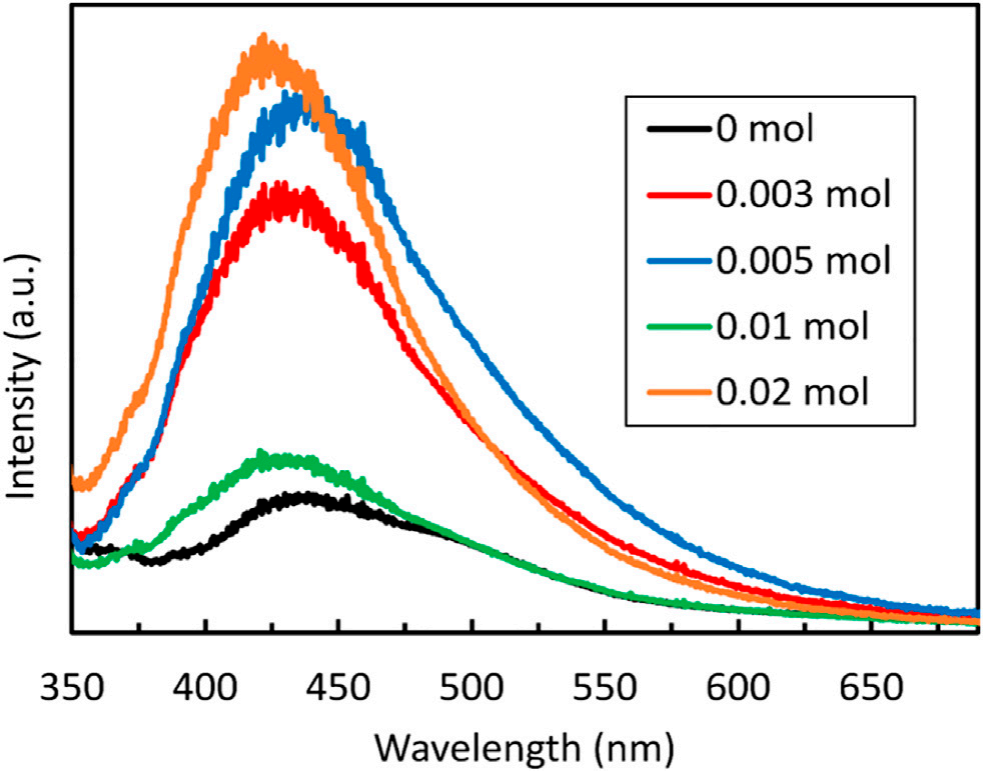
The Photoluminescence (PL) spectra of ZnS synthesized with different amounts of NaBH_4_.

Photoluminescence (PL) emission provides indication of charge separation where high intensities indicate quick recombination of photogenerated carriers. From Figure 6, it can be seen that NaBH_4_-free ZnS has the lowest intensity of photoluminescence emission, indicating that the photogenerated carriers are not quickly recombined. The PL intensities increased gradually with the addition of NaBH_4_ until the concentration of 0.005 mol of NaBH_4_ was reached, after which they drop significantly when 0.01 mol of NaBH_4_ was added. However, when 0.02 mol of NaBH_4_ was added the intensity increased significantly again. The drop in the PL intensity for the sample prepared with 0.01 mol NaBH_4_ indicates reduced recombination of charge carrier suggesting better photocatalytic activity. This implies that the aforementioned S vacancies could become centres for the recombination of photogenerated electrons and holes when present in excessive amount as 0.01 mol is a percolation threshold found for the PL intensity of the ZnS samples indicated in earlier works (Wang et al., 2015b). High PL intensities on the other hand suggest few defects and fast recombination of photogenerated charge carriers, resulting in decreased photocatalytic activity. This finding is consistent with the results of the photocatalytic degradation and hydrogen production of these ZnS samples as is further outlined below.

### 3.2 Computational Modelling

#### 3.2.1 Bulk ZnS

**FIGURE 7:**
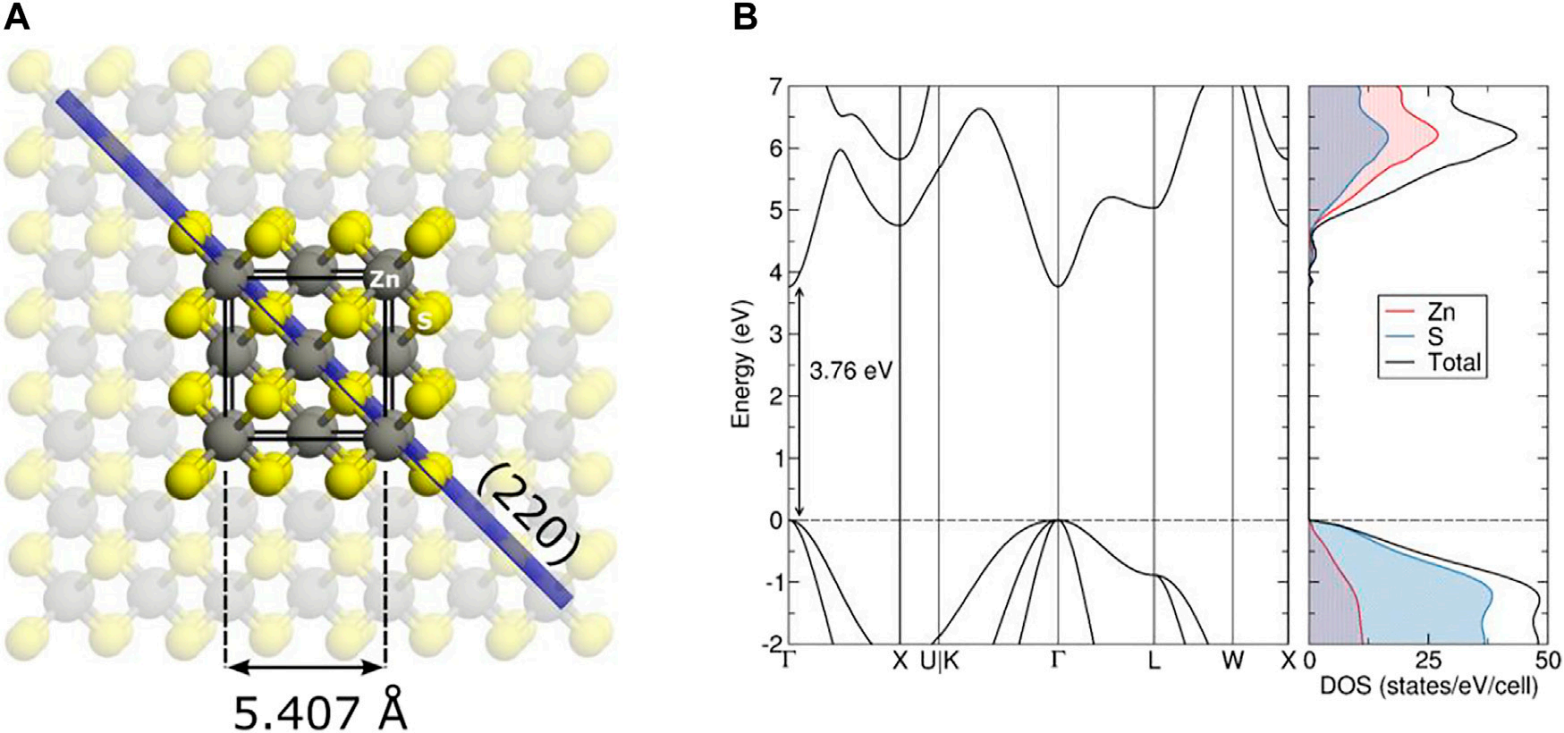
Crystal structure of bulk ZnS sphalerite shown on the **(A)**. The unit cell is indicated with black lines, the (220) crystallographic plane with the blue coloured plane, while zinc and sulphur atoms are coloured grey and yellow, respectively. On the **(B)**, the electronic band structure together with the atom-projected densities of state are shown. The dashed line indicates the highest occupied state.

To further elucidate the structural properties of ZnS, DFT calculations were employed. The relaxed crystal structure of ZnS in the sphalerite phase is shown in Figure 7. The fully relaxed geometry yields a lattice parameter of 5.407 Å, which is in excellent agreement with the experimental value of 5.409 Å measured by Skinner (Skinner, 1961). ZnS sphalerite crystallizes in a high-symmetry cubic structure with the space group ( $F\bar{4}3m$ , number 216) where each Zn atom is found in a tetrahedral environment of surrounding S atoms. The same applies for the S atoms, where each S is surrounded by four Zn atoms.

The calculated electronic band structure and density of state (DOS) for ZnS sphalerite are shown on the right of Figure 7. The calculated band gap reads 3.76 eV located at the Γ point, which is in reasonable agreement with the experimentally observed value of 3.55 eV outlined earlier. The top of the valence band (VB), up to −2 eV on an energetic scale, is primarily comprised of Zn *d* states weakly mixed with S *p* states. The conduction band (CB) originates from empty Zn and S states mixed almost equally. For comparison, the band gap calculated with the B3LYP global hybrid functional reads 3.91 eV and shows no significant improvement when describing the electronic properties of ZnS.

#### 3.2.2 Bulk Vacancies

Single sulphur and zinc vacancies were introduced into bulk ZnS to probe the potential effect on the electronic structure and subsequently the absorption in the UV visible part of the electromagnetic spectrum. A 3 × 3 × 3 supercell was created from the relaxed bulk ZnS structure, to ensure that there is minimal interaction between the periodically repeated defect images, located at least 16 Å apart in each crystallographic direction. Single individual sulphur and zinc vacancies were then introduced separately in the supercell (labelled $V_{S}^{bulk}$ and $V_{Zn}^{bulk}$ ).

Removing a zinc atom from the lattice leaves four dangling bonds behind from the nearest neighbouring phosphorus atoms with mainly *p* orbital character. Upon relaxation, the sulphur ions are symmetrically repelled off the vacant site, with their bond length being contracted by roughly 2% $\left(d_{Zn-S|Zn-vac|}^{bulk}=2.294\;Å\right)$ . The projected DOS of the fully relaxed geometry (not reported for brevity) reveal doubly occupied states present at approximately 0.3 eV above the VB maximum of the pristine bulk material. These states originate predominantly from states localized on sulphur atoms in the immediate proximity of the vacancy and to a lesser extent from states of zinc atoms surrounding the site. However, even under S-rich conditions, the formation energy $V_{Zn}^{bulk}$ is more than 3.5 eV, making this defect difficult to materialize.

In contrast, $V_{S}^{bulk}$ is energetically more favourable, costing little more than 2 eV to form (under Zn-rich conditions). Sulphur is nominally found in the -2 charge state, leaving two electrons behind when removed from the lattice. These electrons have only the surrounding high energy Zn *p* and *d* states to move into, thus forming a doubly occupied localized state merging the top of the VB as well as acceptor states at the bottom of the CB. The electronic band gap remains mostly unaltered (Table 2).

**TABLE 2:** Calculated defect formation energies (E^f^) together with the electronic band gap values (E_g_, estimated from singe particle Kohn-Sham eigenvalues) of sulphur and zinc vacancies present in the bulk as well as (220) surface of ZnS. The up (down) arrows represent spin up (down) states. All formation energies correspond to neutral defects and were obtained using HSE06.

Defect	E^f^ (eV)		E_g_ (eV)
	Zn-rich/S-poor	Zn-poor/S-rich	
$V_{S}^{bulk}$	2.06	4.37	3.79
$V_{Zn}^{bulk}$	5.84	3.53	3.55
$V_{S}^{surf}\left(top\right)$	1.19	3.50	3.22
$V_{Zn}^{surf}\left(top\right)$	4.17	1.87	2.33 ( ↑ )/3.92 ( ↓ )
$V_{S}^{surf}\left(sub\right)$	1.64	3.95	3.56
$V_{S}^{surf}\left(mid\right)$	1.70	4.01	3.45
$V_{S-double}^{surf}\left(top\right)$	2.13	6.74	3.19

#### 3.2.3 Surfaces

The (220) surface was chosen to model adsorption effects as it results in a non-polar termination and is observed as one of the dominant planes in the outlined XRD analysis. A 1.7 nm thick slab was cleaved from the relaxed bulk of ZnS along the (220) direction and expanded into a 3 × 2 supercell in the a-b plane for subsequent adsorption as well as analysis of occurring surface defects. This results in a simulation cell where periodic images are located at least 10.8 Å apart.

The initially cut slab terminates with a layer of Zn and S atoms residing in the same plane. Upon relaxation, the topmost Zn atoms move towards the slab as a consequence of the missing tetrahedron corner S atom, resulting in an increased Coulomb attractive force towards the remaining three S atoms. In the final relaxed geometry, the top Zn atom has transformed from a tetrahedral to an asymmetric trigonal planar coordination. The undercoordinated surface S atoms are pushed further out and define the surface termination. The surface Zn atom forms two bonds with the most prominent surfaces S atoms $\left(d_{Zn-S|top}^{surf}=2.274\;Å\right)$ and one with the first subsurface layer S atom $\left(d_{Zn-S|sub}^{surf}=2.296\;Å\right)$ , all three being shorter compared to the starting bulk value $\left(d_{Zn-S}^{bulk}=2.342\;Å\right)$ . The top S atom binds the aforementioned two surface Zn atoms and one subsurface Zn atom at a distance that almost recovers the bulk value $\left(d_{S-Zn|sub}^{surf}=2.332\;Å\right)$ . The calculated surface energy of ZnS(220) is just 0.571 J/m^2^, explaining from an energetic perspective its prominent presence in the observed morphologies. As a result of the structural rearrangement, the electronic band gap of the (220) surface is calculated at 3.88 eV, being around 0.1 eV larger than the initial bulk band gap.

#### 3.2.4 Defects on Surfaces

**FIGURE 8:**
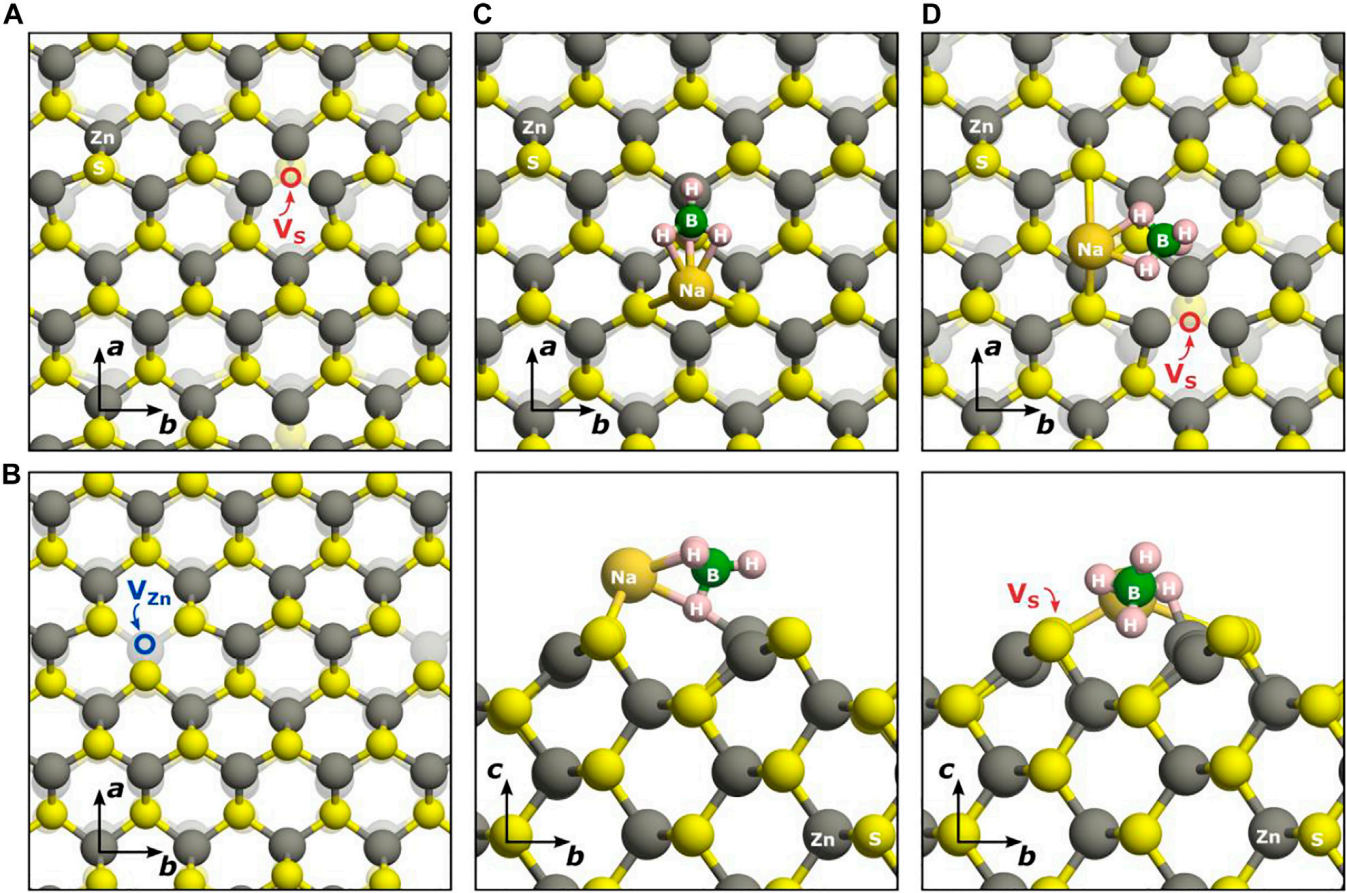
Crystal structure of defects and adsorbates at the ZnS (220) surface: single sulphur vacancy **(A)**, single zinc vacancy **(B)**, adsorption of NaBH_4_ on the pristine (220) surface **(C)**, adsorption of NaBH_4_ on a (220) surface where a single sulphur vacancy is present **(D)**.

**FIGURE 9:**
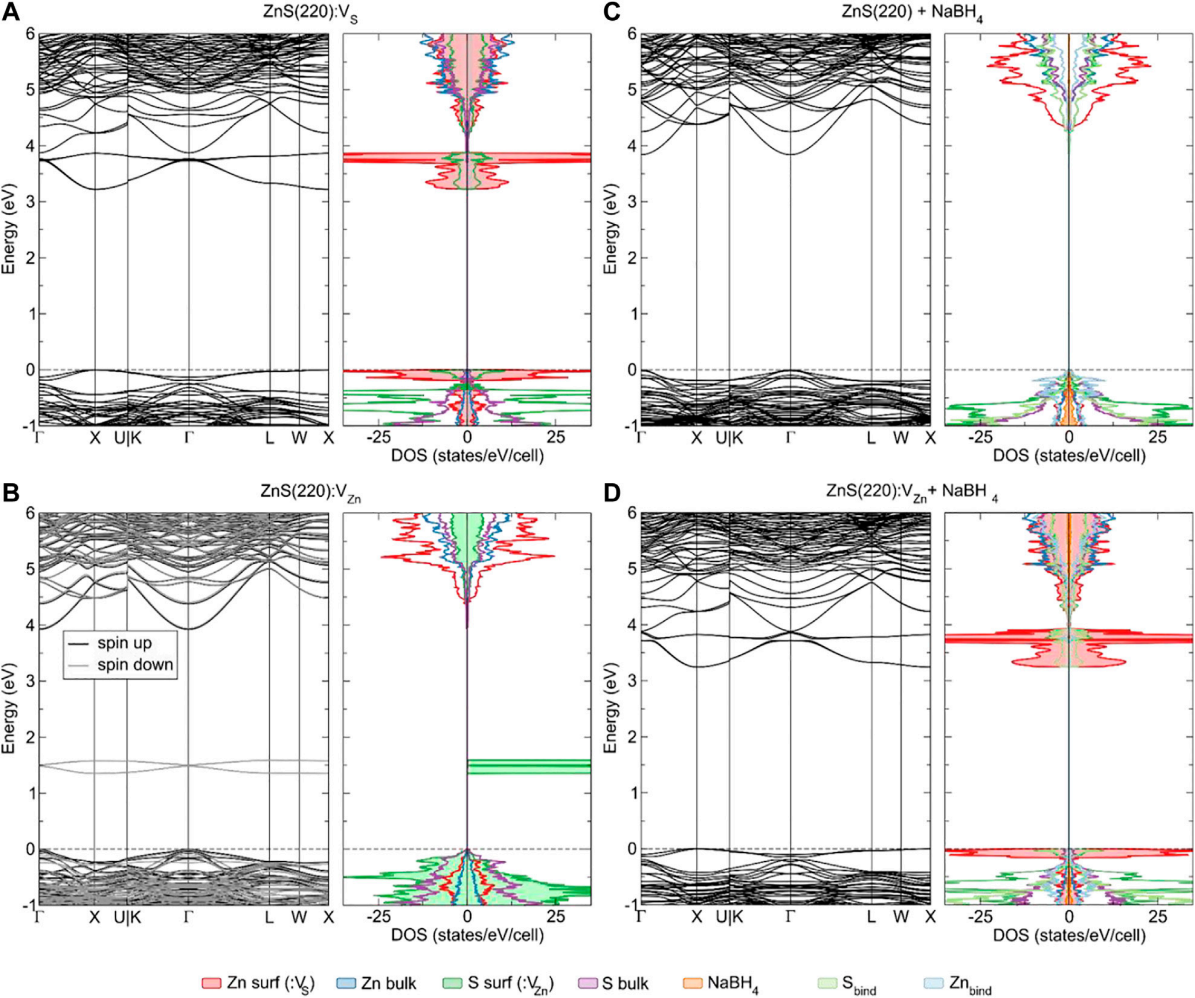
Electronic band structure and densities of states of: ZnS(220):V_S_ **(A)**, ZnS (220):V_Zn_ **(B)**, ZnS (220) + NaBH_4_ **(C)**, ZnS (220):V_S_ + NaBH_4_ **(D)**. The dashed lines indicate the highest occupied state.

To gain further insight into the properties of the (220) surface, single vacancies were introduced into the slab and their energetics evaluated. The relaxed geometries of a single sulphur as well as zinc vacancy present in the topmost surface layer are depicted on the right of Figure 8. As a results of the induced zinc vacancy, the sulphur moves away from the top zinc by 0.02 Å at the same time shortening the distance to the subsurface zinc by 0.02 Å. The spin density difference (from a calculated Mulliken population analysis) reveals two localized electrons spread between the two top sulphur and one subsurface sulphur atom, with values of $△S_{top}=0.86\;e^{-}$ and $△S_{subsurf}=0.21\;e^{-}$ . These states are clearly visible in the partial DOS (Figure 9) as well as two defect hole states localized on the aforementioned sulphur atoms, centred at around 1.5 eV above the VB maximum. As a result of the modified coordination environment in which the top Zn atom is found, the $V_{Zn}^{surf}\left(top\right)$ formation energy has decreased by almost 2 eV, calculated at 1.87 eV which is much easier to achieve compared to the bulk vacancy.

The single sulphur vacancy present at the surface behaves similarly to its bulk analogue. The electrons remaining at the site when sulphur is removed are localized at the nearest neighbouring zinc atoms, forming occupied states at the top of the VB as well as empty acceptor states at the bottom of the CB. In the relaxed geometry, the zinc atoms surrounding the vacancy move towards the empty site (Figure 8A), elongating the zinc-(top)-sulphur bond by 0.11 Å and the zinc-(subsurface)-sulphur bond by 0.06 Å. This rather large geometry change induces a lowering of the band gap by almost 0.6 eV compared to the bulk vacancy. As a consequence of the incomplete tetrahedral coordination, the formation energy of $V_{S}^{surf}\left(top\right)$ is only 1.19 eV under Zn-rich condition, which is almost half of the value required to extract a sulphur from the bulk of the material. However, the formation energy increases significantly when trying to remove any sulphur below the top layer (values listed in Table 2) and the band gap increases towards a value found in the bulk of ZnS.

A double sulphur vacancy has been tested at the top of the ZnS (220) surface as well. Due to the increased computational cost and for easier comparison, the two vacancies were introduced in the same supercell where the single vacancy was modelled earlier. This way, the vacancies are set apart by only 6.5 Å, yet the relaxed geometry as well as electronic structure reveal little to no overlap between the defect wavefunctions. The calculated band gap of the surface containing $V_{S-double}^{surf}\left(top\right)$ is 3.19 eV, which is almost identical to the band gap of 3.22 eV for the surface containing $V_{S}^{surf}\left(top\right)$ . The projected DOS reveal doubling of the states identified in the case of the single sulphur vacancy, with neither new donor nor acceptor states found in the electronic structure of the surface. The formation energy of the two vacancies is calculated at 2.13 eV, indicating that the (220) surface of ZnS can readily accommodate multiple vacancies in its topmost layer.

#### 3.2.5 Adsorption on Pure and Defective Surfaces

The adsorption of sodium borohydride on top of a pristine ZnS(220) surface results in one binding mode, regardless of the initial position of the NaBH_4_ molecule. The molecule attaches itself such that Na bridges two S atoms and one of the H atoms from the $BH_{4}^{-}$ ion binds to the most prominent Zn atom. The electronic structure of the surface with the adsorbed NaBH_4_ is shown in Figure 9C. NaBH_4_ is an insulating molecule with a band gap of 9.74 eV (calculated at HSE06 level) and does not induce any defect states in the band gap of the pristine ZnS(220) surface, further confirming what was observed in the XPS analysis earlier. The molecule binds strongly to the surface, releasing 1.34 eV in energy upon adsorption. The changes in bond lengths are reported in Table 3, with the strongest increase in spatial separation noted between the $\;Na^{+}$ and $BH_{4}^{-}$ ions.

**TABLE 3:** Calculated adsorption energy of NaBH_4_ bound onto ZnS(220) in its pristine and sulphur deficient form and electronic band gap together with the accompanying bond lengths (values reported in Å).

System	$E_{b}^{\mathrm{CP}}$ (eV)	*E*_*g*_ (eV)	*d* (Na-H)	*d* (Na-B)	*d* (B-H)	*d* (Na-S)	*d* (H-Zn)
NaBH_4_	N/A	9.74	2.179	2.293	1.250	N/A	N/A
			2.182		1.250		
			2.185		1.250		
					1.207		
ZnS(220)+ NaBH_4_	− 1.34	3.84	2.247	2.443	1.224		1.780
			2.309		1.224	2.799	
			2.317		1.269	2.803	
					1.212		
ZnS (220):V_S_ + NaBH_4_	− 1.05	3.25	2.207	2.703	1.225		1.859
			2.266		1.228	2.677	
			3.351		1.273	3.211	
					1.207		

Upon addition of a sulphur vacancy on one of the sites where NaBH_4_ binds to the surface and subsequent geometrical relaxation, the molecule rotates away from the vacant site in order for sodium to find a suitable sulphur to bind to. In this way, NaBH_4_ binds more strongly to the nearest sulphur site, elongating the bond to the nearest Zn and weakly binding the second sulphur atom at only 3.211 Å. As a consequence, the adsorption energy is reduced by almost 0.3 eV compared to the surface without any defects present. The electronic band gap is again not altered compared to the case with only a single vacancy present at the surface, confirming the inability of sodium borohydride to alter the electronic properties of the ZnS(220) surface, regardless of its pristine or defective form.

### 3.3 Photocatalytic Performance

**FIGURE 10:**
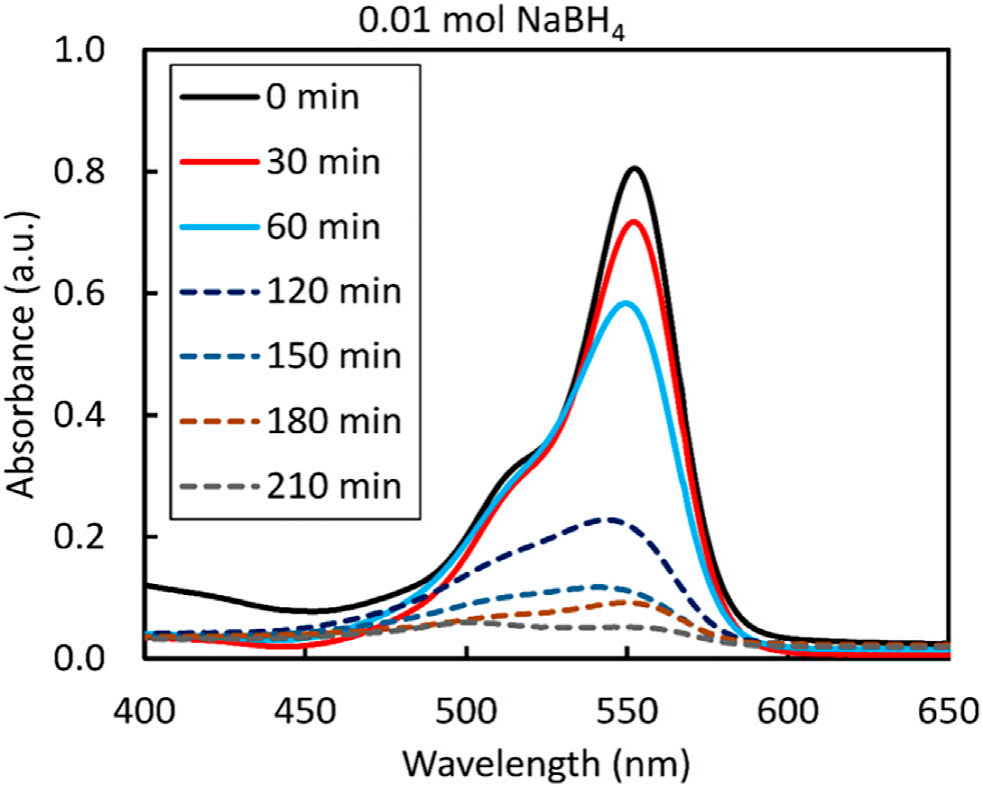
Optical absorption spectra of RhB catalysed by ZnS prepared using 0.01 mol NaBH_4_.

**FIGURE 11:**
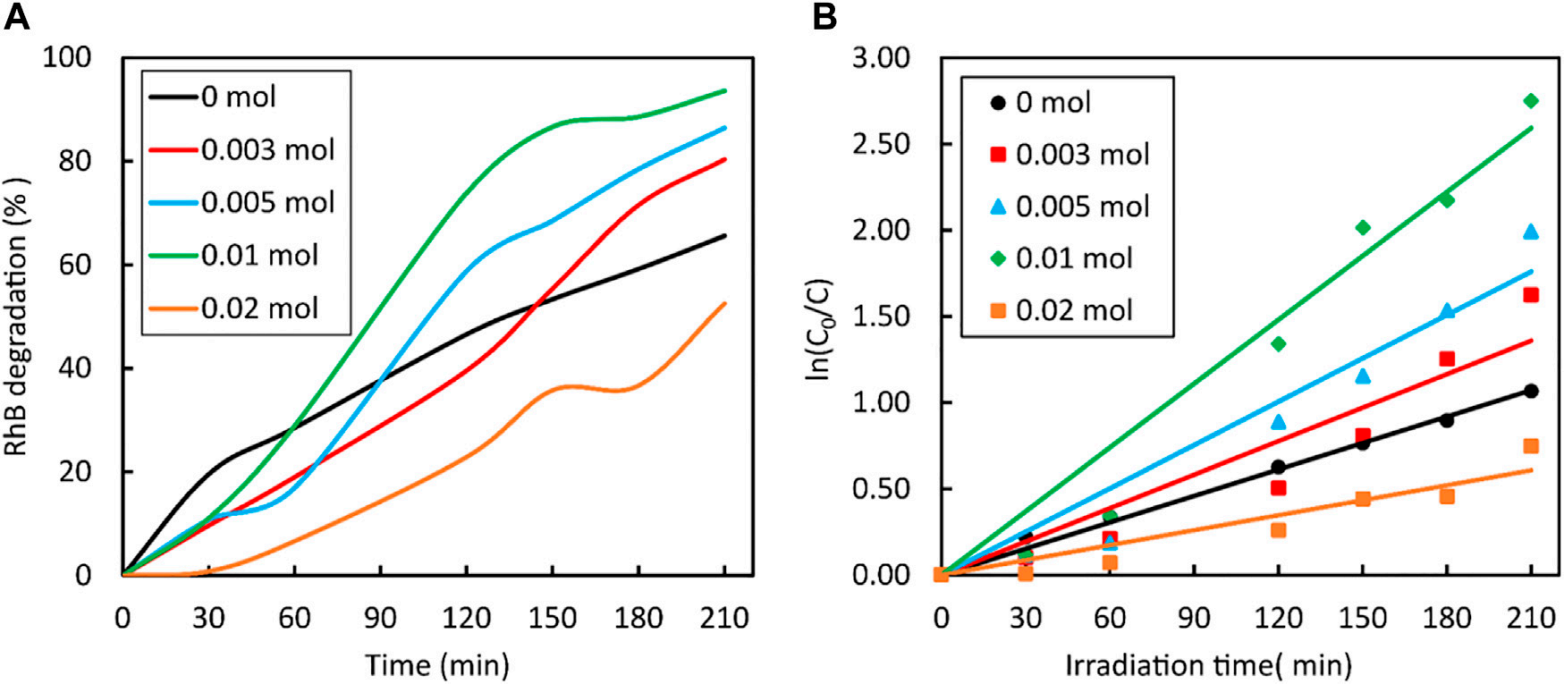
Degradation rate of RhB dye by on the **(A)** and the kinetic curves for the degradation of Rh B dye by ZnS on the **(B)**, both prepared using different amounts of NaBH_4_.

The photocatalytic performance of the synthesized ZnS nanoparticles was evaluated for the degradation of RhB under UV light (*λ*_max_ = 553 nm). After exposure to UV light for 210 min, the intensity of the spectrum decreased sharply, showing no characteristic absorption. This suggests complete decolourisation/degradation of RhB (Figure 10). The degradation efficiency of the ZnS nanoparticles prepared under different reaction conditions (Figure 11A) was calculated according to the equation below, where C_0_ is the initial concentration of RhB solution and C is the concentration of the dye at irradiation time (t).

$$\text{D}\text{e}\text{g}\text{r}\text{a}\text{d}\text{a}\text{t}\text{i}\text{o}\text{n}\;\text{e}\text{f}\text{f}\text{i}\text{c}\text{i}\text{e}\text{n}\text{c}\text{y}\;\left(\%\right)=\frac{C_{0}-C}{C_{0}}\times 100\%.$$

All samples showed activity greater than 50% for the degradation of RhB, which increased with the amount of added NaBH_4_. The ZnS nanoparticles prepared with 0.01 mol NaBH_4_ showed the highest efficiency of 93.60% after 210 min of exposure followed by 0.005 mol (86.39%) and 0.03 mol (80.30%). The degradation of the sample prepared with 0.02 mol NaBH_4_ fell sharply (52.57%) even below that of ZnS not treated with NaBH_4_. The behaviour of these two samples, i.e., the ZnS nanoparticles prepared with 0.01 and 0.02 mol of NaBH_4_, is consistent with the trend observed with PL data.

The reaction kinetics of RhB (Figure 11B) can be explained by the pseudo-first order equation:

$$\ln\left(\frac{C_{0}}{C}\right)=kt,$$

where $C_{0}$ is the initial RhB concentration at t = 0 , C is the concentration at reaction time *t*, and *k* the reaction rate constant. The values of the kinetic rate constant of ZnS samples prepared with a varying amount of sodium borohydride are listed in Table 4. The ZnS sample prepared with 0.01 mol NaBH_4_ showed the highest kinetic rate constant, whereas the ZnS sample prepared with 0.02 mol NaBH_4_ exhibited the lowest kinetic rate constant. The results indicate that 0.01 mol of NaBH_4_ was the threshold amount at which maximum photocatalytic degradation of RhB can be attained. The photocatalytic activities of these samples for RhB degradation, evaluated under UV light irradiation, show that the photocatalytic activity increases as the concentration of S vacancies increased, until it reaches its percolation threshold of 0.01 M fraction. It then decreases sharply beyond the percolation threshold value because, when present in excessive amounts, S vacancies destroy the morphologies (visible from the SEM images) of the samples and also act as recombination sites of photogenerated electrons and holes (noted from the PL results).

**TABLE 4:** The kinetic curves for the degradation of RhB dye by ZnS prepared using different amounts of NaBH_4_.

NaBH_4_ (mol)	D (nm)	E_g_ (eV)	Rate Constant (min^−1^)
0.000	12.1	3.54	0.0051
0.003	12.5	3.54	0.0065
0.005	12.4	3.55	0.0084
0.010	13.8	3.55	0.0123
0.020	8.04	3.57	0.0029

**FIGURE 12:**
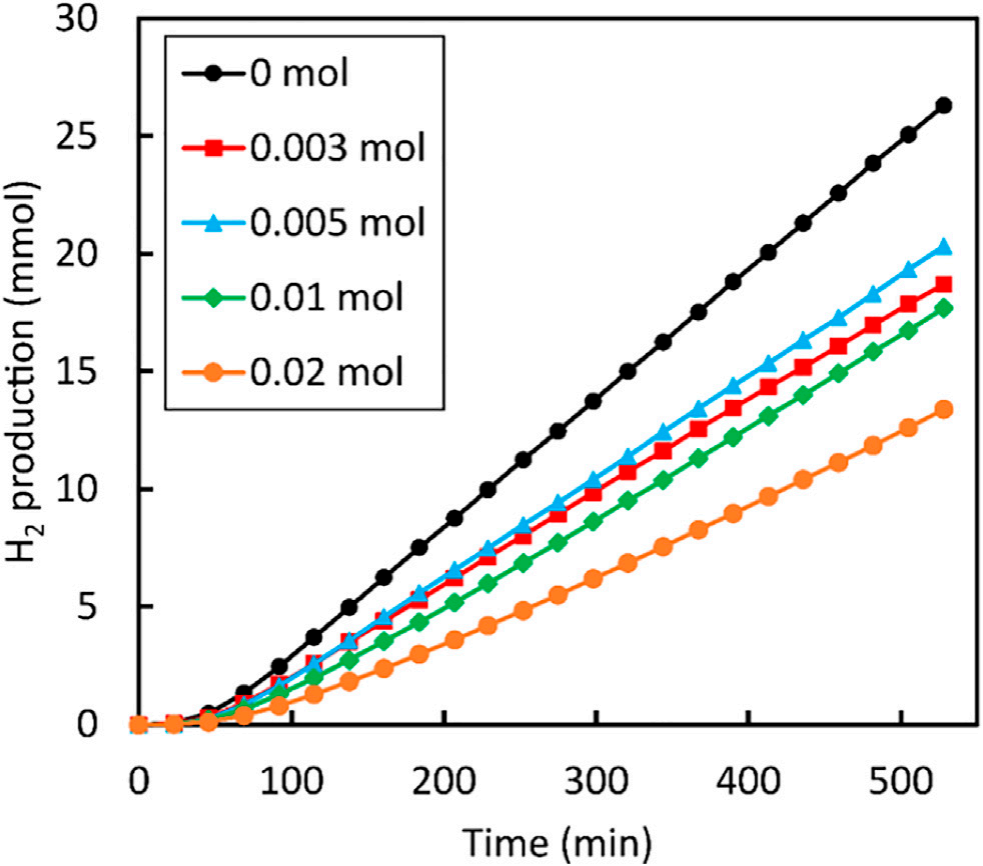
H_2_ production by ZnS nanoparticles prepared using different amounts of NaBH_4_.

When the same ZnS samples were tested for the photocatalytic H_2_ generation, the results did not reflect the trend that was obtained for the dye degradation of RhB. All samples showed activity, but the amount of H_2_ generated from ZnS samples prepared with the addition of NaBH_4_ were all below the value of the sample that was not treated with NaBH_4_ (Figure 12). This suggests that the addition of NaBH_4_ to ZnS nanoparticles prepared in this study did not improve the generation of H_2_, which is in conflict with the results reported by Wang et al. (2015b). The rate of reaction is affected by various factors including concentration of reactants, catalyst dose and light intensity. The starting concentration of Zn and S in NaOH used in this study is 0.292 M. This is potentially different when compared to the work of (Wang et al., 2015b), as their procedure did not specify the amount of Zn and S powders added to NaOH. In addition to this, the current study used a low dosage of the catalyst (0.001 g/ml) compared to 0.005 g/ml used in the already mentioned study. Finally, the lamp that was used in our study is a 100 W LED which is less efficient compared to the 300 W arc Xe lamp used by Wang et al. (2015b).

Furthermore, our band gap analysis reveals that these ZnS samples are not photo-responsive to light with wavelength greater than 350 nm. Moreover, the S vacancies which are claimed to generate mid-gap defect states in ZnS from earlier studies are not found in the present work and indicate a possible overestimation of defect levels as a result of using a semi-local functional compared to the hybrid one and not explicitly considering surface defects while rather extrapolating values obtained from the bulk.

## 4 Conclusion

ZnS with various molar fractions of NaBH_4_ in a ZnS matrix were successfully fabricated using a facile one pot-hydrothermal method, and their structural and photocatalytic properties were clarified. Increasing the NaBH_4_ molar fractions from 0.005 to 0.01 mol, which increases the number of S vacancies, enhances the photocatalytic degradation of the RhB dye due to the photogenerated carriers which do not quickly recombine. Based on the relationship between the molar fraction of NaBH_4_ and the photocatalytic activities of the ZnS samples, the percolation threshold of the ZnS/NaBH_4_ system was found at a NaBH_4_ molar fraction of 0.01 mol. It was elucidated from XPS, SEM, and PL observations that the decrease in photocatalysis at larger NaBH_4_ concentrations was attributed to a surplus of S vacancies formed, a change in the morphology of the ZnS samples, and the separation efficiency of photogenerated charge carries in ZnS samples. DFT studies of sulphur vacancies present in the bulk as well as surface of ZnS show favourable formation energies as well as band gap lowering of ZnS. However, the resulting band gap is still not found responsive to the visible part of the UV spectrum and further reduction to achieve so are necessary. The adsorption of NaBH_4_ atop the (220) surface of ZnS was found to be energetically stable and no defect levels were found in the otherwise pristine band gap, neither from XPS nor DFT characterizations. Ultimately, it is still a challenge to achieve ZnS photocatalysts with a high value of overall quantum efficiency due to rapid recombination rates of charge carriers and further optimizations are required to exhaust their full potential.

## Data Availability

The original contributions presented in the study are included in the article/Supplementary Material, further inquiries can be directed to the corresponding author.
